# Application of a new body surface-assisting puncture device in percutaneous transforaminal endoscopic lumbar discectomy

**DOI:** 10.1186/s12891-022-05985-4

**Published:** 2022-12-06

**Authors:** Xincheng Fan, Qiting He, Chaofan Yi, Wei Zhao, Derui Xu, Guoqing Peng, Feng Liu, Lei Cheng

**Affiliations:** 1 Department of Orthopedic Surgery, Cheeloo College of Medicine, Qilu Hospital of Shandong University, Shandong University, 107 Wenhuaxi Road, 250012 Jinan, Shandong China; 2 Department of Orthopedic Surgery, The Affiliated Taian City Central Hospital of Qingdao University, 271000 Taian, Shandong China

**Keywords:** Percutaneous transforaminal endoscopic lumbar discectomy, Lumbar disc herniation, Body surface-assisting puncture device, Puncture, X-ray fluoroscopy

## Abstract

**Background:**

Accurate puncture and localization are critical for percutaneous transforaminal endoscopic lumbar discectomy surgery. However, several punctures are often required, followed by X-ray fluoroscopy, which can increase surgical risk and complications. The aim of this study was to demonstrate a new body surface-assisting puncture device that can be used in percutaneous transforaminal endoscopic lumbar discectomy and to assess its clinical effectiveness.

**Methods:**

Three hundred and forty-four patients were treated with percutaneous transforaminal endoscopic lumbar discectomy surgery in the Spinal Surgery Department of Taian City Central Hospital, China, between January 2020 and February 2022. Of these, 162 patients (the locator group) were punctured using a body surface-assisting puncture device while and 182 patients (the control group) were punctured using the traditional blind puncture method. The number of punctures, radiation dose during X-ray fluoroscopy, operation time, and surgical complications were compared between the two groups.

**Results:**

The average number of punctures was 2.15 ± 1.10 in the locator group which was significantly lower than that in the control group (5.30 ± 1.74; *P < 0.001*). The average X-ray fluoroscopy radiation dose in the locator group was significantly lower at 2.34 ± 0.99 mGy, compared with 5.13 ± 1.29 mGy in the control group (*P < 0.001*). The mean operation time was also significantly less in locator group (47.06 ± 5.12 vs. 62.47 ± 5.44 min; *P = 0.008*). No significant differences in surgical complications were found between the two groups (*P > 0.05*).

**Conclusion:**

The use of a new body surface-assisting puncture device in percutaneous transforaminal endoscopic lumbar discectomy surgery can significantly reduce the number of punctures and X-ray fluoroscopy radiation dose, as well as shortening the operation time, without increasing surgical complications. This device is cheap, easy to operate, and suitable for all hospitals and spine surgeons, especially for small hospitals, with also no extra costs for patients.

## Background

Percutaneous transforaminal endoscopic lumbar discectomy, also known as percutaneous endoscopic lumbar discectomy (PELD), is widely used in the treatment of lumbar disc herniation (LDH) as it has the advantages of less tissue damage, less bleeding, less risk and complications, and rapid recovery after surgery [1]. Furthermore, the operation is performed under local anesthesia, reducing the cost of surgery [2, 3]. Accurate puncture and localization are critical in PELD surgery, and often more than a few punctures are required, followed by X-ray fluoroscopy, which can cause tissue and nerve damage [4, 5]. It is often difficult to reduce the number of punctures with accurate puncture localization, especially for junior surgeons. Thus, increased numbers of punctures and increased use of X-ray fluoroscopy are often unavoidable, increasing surgical risk and complications and leading to a steep learning curve for young doctors in PELD surgery [6, 7]. The doctors at Taian City Central Hospital invented an auxiliary puncture instrument to solve the problem of accurate puncture localization (body surface-assisting puncture device). This study aimed to investigate the role of the auxiliary puncture instrument in PELD surgery and to support its clinical application in PELD surgery.

## Materials and methods

### General Information

The study included 344 patients diagnosed with lumbar disk herniation (LDH) at the Spinal Surgery Department of Taian City Central Hospital, China, between January 2020 and February 2022. The patients were randomly assigned to two groups: the locator group in which the body surface-assisting puncture device was used (male:female = 86:76, average age: 40.01 ± 6.14); the control group who received the traditional blind puncture method (male:female = 83:99, average age: 39.85 ± 5.42). The Taian Central Hospital Ethics Committee approved this research, and all patients provided informed consent for surgery. All surgeries were performed by the same experienced spine surgeon using PELD technology.

Inclusion criteria: (1) patients with unilateral, single-level LDH; (2) patients that agreed to PELD surgery; (3) patients without any other serious diseases. Exclusion criteria: (1) patients with multiple-level LDH; (2) patients suffering from lumbar spondylolisthesis, lumbar instability, spinal stenosis, and other related spinal diseases; (3) patients with mental diseases or mental dysfunction.

Patients were randomly assigned to the two groups using a random number table method. Patients in the locator group were punctured using a body surface-assisting puncture device while those in the control group were punctured using the traditional blind puncture method.

### The structure schematic diagram and appearance structure of body surface-assisting puncture device

**Fig. 1:**
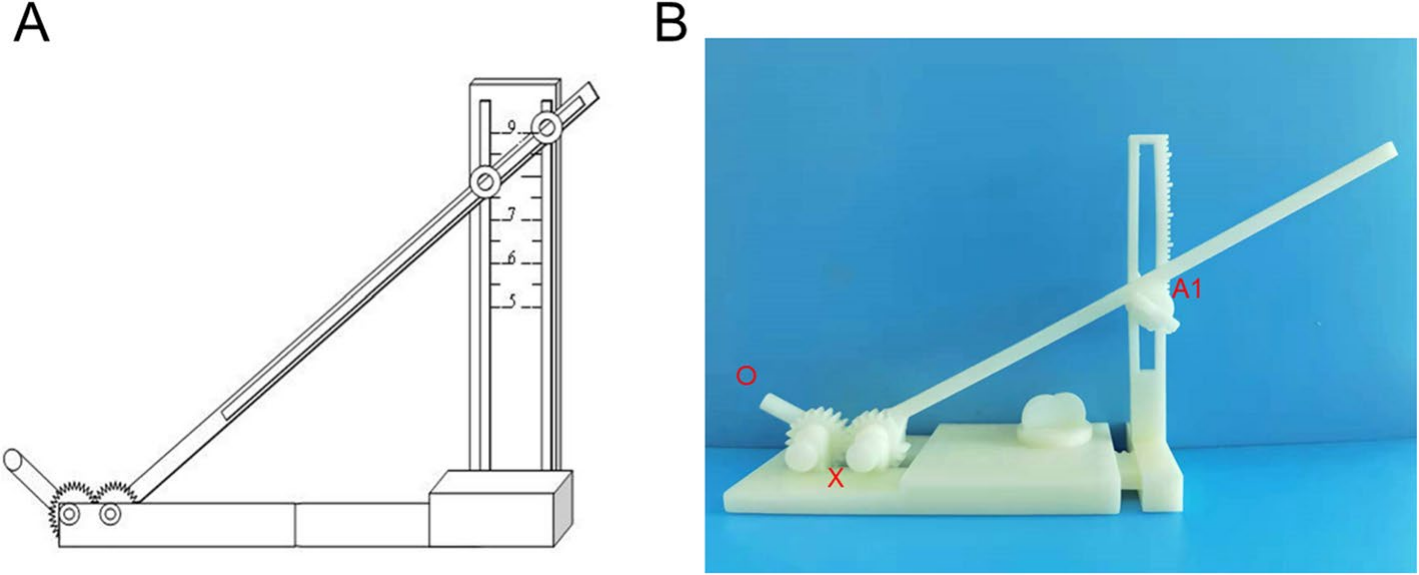
Schematic diagram of the structure and appearance structure of body surface-assisting puncture device.** A** The structure schematic diagram of body surface-assisting puncture device. **B** The appearance structure of 3D-printed body surface-assisting puncture device

Figure 1A depicts a schematic diagram of the structure of the device, while the image of the 3D-printed body surface-assisting puncture device is shown in Fig. 1B. OX represents a puncture tube, and A1 denotes the adjustable height of the body surface-assisting puncture device. The angle of the puncture pipe OX can be automatically and symmetrically adjusted using a gear.

### Geometric schematic diagram of body surface-assisting puncture device guided puncture path

**Fig. 2:**
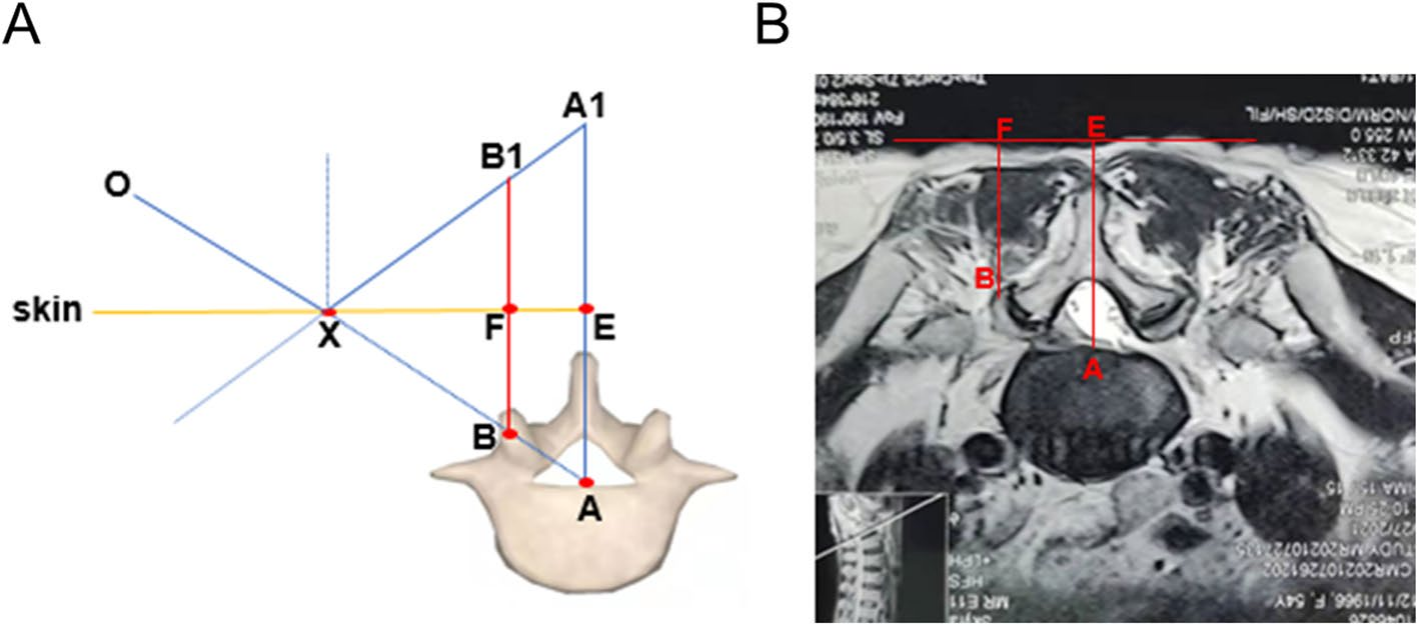
Geometric schematic diagram of locator-guided puncture path.** A** The geometric schematic diagram of locator-guided puncture path. **B** The method of AE distance measurement on MRI cross section

Figure 2A shows the geometric schematic diagram of the body surface-assisting puncture device guided puncture path; the geometry principle refers to a symmetry of isosceles triangles. Point A is the midpoint of the posterior upper margin of the lower vertebral body of the operation segment, and point B is the apex of the superior articular process. Points A1 and B1 are symmetric points A and B, respectively; points E and F are the skin projections of points A and B. The intersection X of lines AB and A1B1 represents the skin puncture point, whereas line OA represents the puncture route. The key to using the body surface-assisting puncture device is to measure the vertical distance (line AE) between the midpoint of the posterior upper margin of the operation segment’s lower vertebral body and the skin. Line A1E is equidistant from line AE and represents the height at which the body surface-assisting puncture device must be adjusted. A picture-archiving and communication system was used to calculate the AE distance on the transverse section of CT or MRI (Fig. 2B).

### Surgical Procedures

**Fig. 3:**
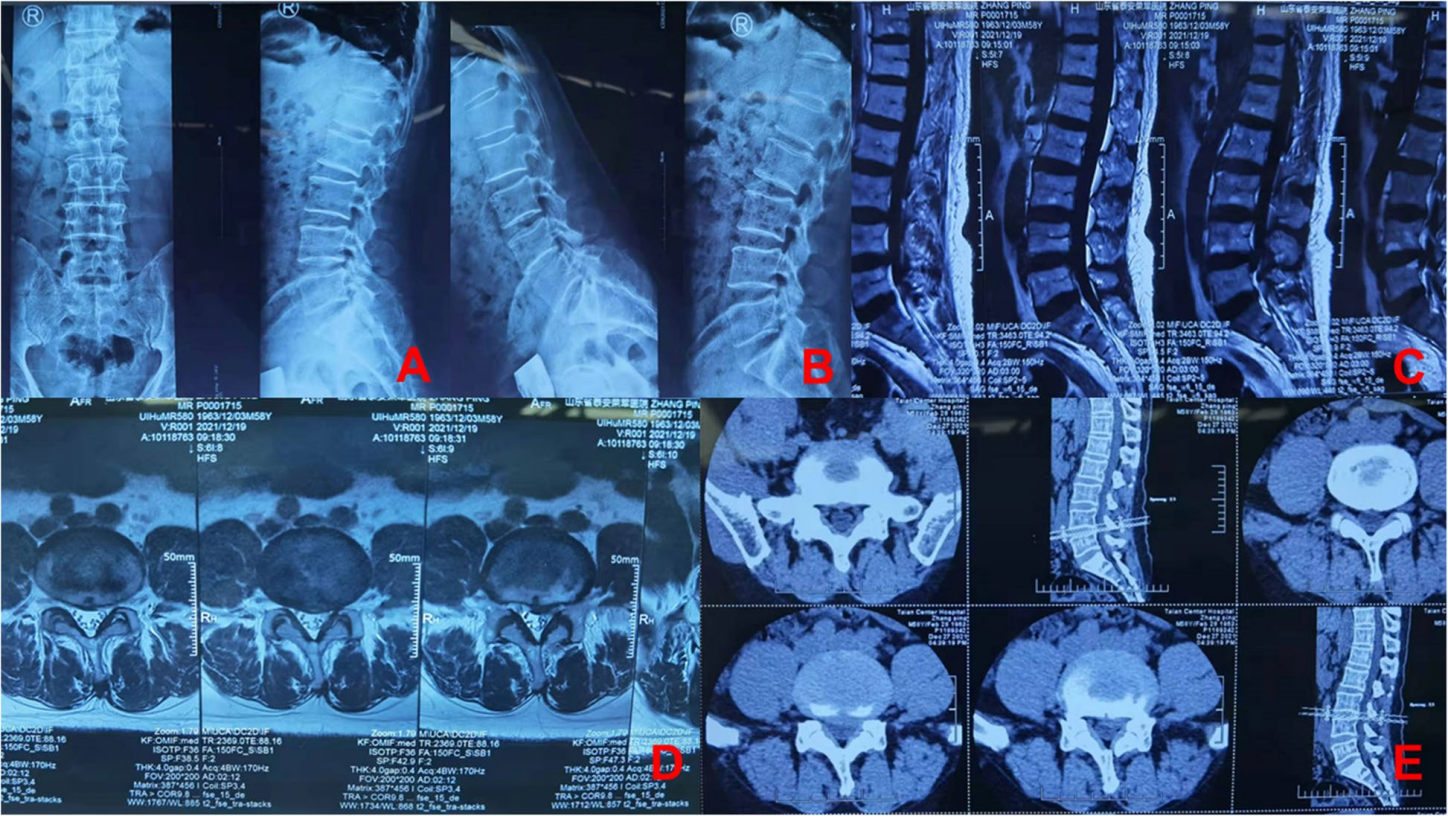
Imaging data of one patient with lumbar 4/5 disc herniation.** A**, **B** Preoperative X-ray. **C**, **D** Preoperative MRI. **E** Preoperative CT

**Fig. 4:**
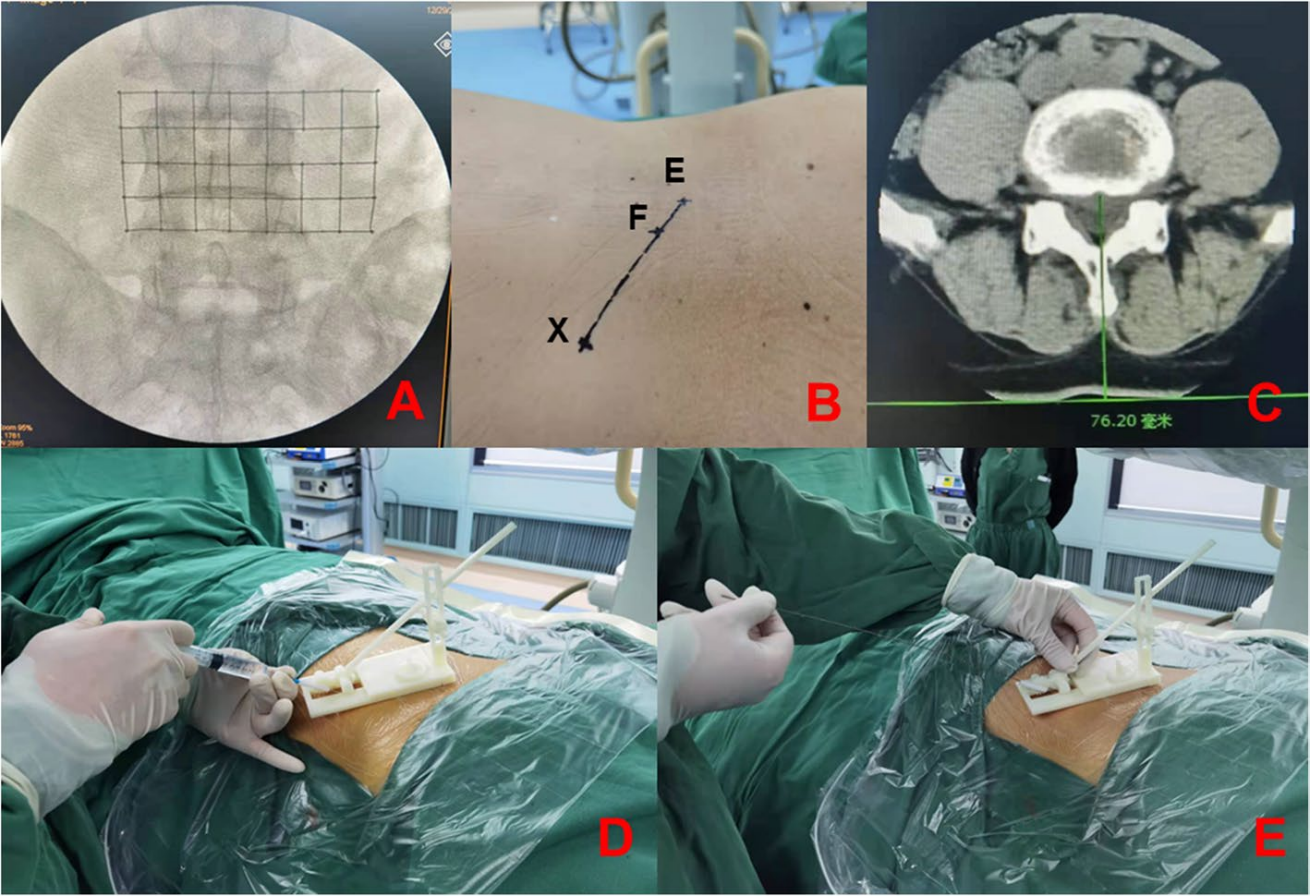
Application of the body surface-assisting puncture device during surgery.** A**, **B **The iron positioning grid was used to locate the puncture point. **C** To measure the vertical distance between the midpoint of the posterior upper margin of the lower vertebral body to skin on the CT cross-section. **D **Adjust the height of the body surface-assisting puncture device according to the measured distance to determine the puncture path, then the anesthesia was infiltrated layer by layer. **E **Use the needle to puncture to the target point through the body surface-assisting puncture device

All patients were placed in the prone position and local infiltration anesthesia was used. We selected a case of lumbar 4/5 disc herniation (Fig. 3) to describe the use of the body surface-assisting puncture device. Under the C-arm X-ray machine, the skin projection point E of the posterior upper margin of the lower vertebral body and the skin projection point F of the apex of the superior facet were located using the iron positioning grid, thereby determining the puncture point X (Fig. 4A, B).

**Fig. 5:**
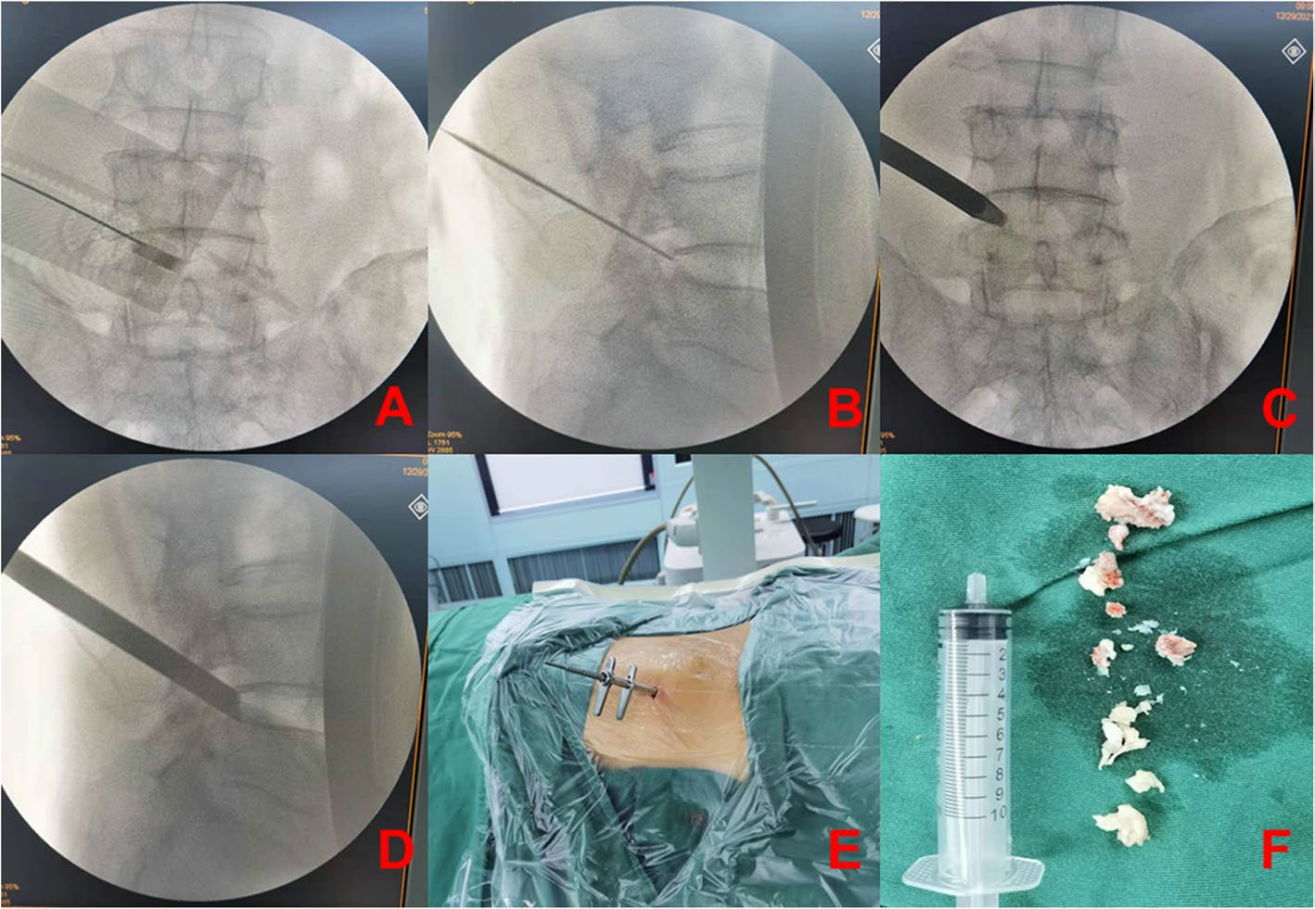
The PELD working channel in place to complete the surgery. **A-E **Place the cannula step by step after confirming the correct position under X-ray fluoroscopy. **F** Lumbar 4/5 herniated disc tissue was removed during surgery

According to the preoperative CT/MR measurement, the vertical distance between the midpoint of the posterior upper margin of the lower vertebral body to the skin was calculated in the locator group (Fig. 4C). The sterile body surface-assisting puncture device was then placed on the surgical site. The puncture point and the edge of the body surface-assisting puncture device coincided with points X and E, adjusting the height of the body surface-assisting puncture device based on the measured distance. The angle of the punctured tube was then automatically and symmetrically adjusted by gears, and local infiltration anesthesia was administered (Fig. 4D). The needle was then used to puncture the target point via the guide tube (Fig. 4E). After confirming the correct position with X-ray fluoroscopy, the cannula was placed step by step (Fig. 5A-E), and the protruding intervertebral disc tissue was removed (Fig. 5F).

For the control group, the needle was used to puncture the correct target point through puncture point X under repeated X-ray fluoroscopic confirmation. The cannula was then placed step by step to remove the protruding intervertebral disc tissue.

### Observation indicators

In each group, the general clinical data of patients, intraoperative puncture times, fluoroscopic times, and operation times were recorded.

### Statistical analysis

IBM SPSS Statistics 20.0 was used for data analysis. The data are represented as means and standard deviations (X ± S). Independent-sample t-tests were used to analyze the differences between the two groups, while chi-square tests were used to to analyze and compare the counting data of the two groups. *P < 0.05* indicated that the difference was statistically significant.

## Results

### Demographics

No significant differences were observed in age, body mass index, and surgical level between the two groups (*P > 0.05*) (Table 1).

**Table 1:** Comparison of preoperative general conditions of 344 patients undergoing PELD surgery

	Locator group	Control group	*P*-value
Male: Female	86:76	83:99	*0.524*
Age	40.01 ± 6.14	39.85 ± 5.42	*0.805*
BMI	24.03 ± 1.21	24.08 ± 1.18	*0.709*
Surgical segment			
L1/2	1	2	*0.989*
L2/3	3	4	*0.897*
L3/4	6	7	*0.799*
L4/5	69	92	*0.264*
L5/S1	83	77	*0.354*
Total	162	182	

### The body surface-assisting puncture device can significantly reduce the number of punctures and radiation dose during X-ray fluoroscopy

The average number of punctures in the locator group was significantly lower than that in the control group (*P < 0.001*). The locator group had a considerably lower radiation dose during X-ray fluoroscopy than the control group (*P < 0.001*). The operation time of the locator group was shorter than that of the control group, with a significant difference between the two groups (*P = 0.008*) (Table 2).

**Table 2:** Comparison of surgical procedures in 344 patients undergoing PELD surgery

	Locator group (*n* = 162)	Control group (*n* = 182)	*p* value
Puncture number (times)	2.15 ± 1.10	5.30 ± 1.74	*< 0.001*
Radiation dose (mGy)	2.34 ± 0.99	5.13 ± 1.29	*< 0.001*
Operation time (min)	47.06 ± 5.12	62.47 ± 5.44	*0.008*

### There were no significant differences in surgical complications between the two groups (*P* < 0.05)

There was one case of nerve root injury in the locator group, where the adventitia of the nerve root was ruptured during the removal of the herniated disc. In the control group, there were two cases of nerve root injury due to puncture localization. Following the surgery, one case of intraspinal hematoma occurred in each group. One case of surgical site infection occurred in the control group after the operation, which was caused by the patient’s advanced age, diabetes mellitus, and long operation time; there were no cases of postoperative infection in the locator group. The control group suffered one case of intestinal injury due to an incorrect puncture angle; the locator group performed well with no intestinal injury. LDH recurrence occurred in three cases in the locator group and four cases in the control group. Overall, there were no statistically significant differences in surgical complications between the two groups (*P > 0.05*) (Table 3).

**Table 3:** Comparison of surgical complications in 344 patients undergoing PELD surgery

	Locator group (*n* = 162)	Control group (*n* = 182)	*p* value
Nerve damage (cases)	1	2	*> 0.005*
Intraspinal hematoma (cases)	1	1	*> 0.005*
Surgical site infection (cases)	0	1	*> 0.005*
Intestinal damage (cases)	0	1	*> 0.005*
LDH recurrence (cases)	3	4	*> 0.005*

## Discussion

LDH is a common disease requiring surgery in 20% of cases, and minimally invasive surgery for LDH is widely accepted [8]. PELD, as a mature minimally invasive surgical technique, has several advantages in terms of clinical effect and prognosis, which is why it is becoming increasingly popular among doctors and patients. Traditional open surgery for LDH treatment is reported to result in a recurrence rate of 5–15% [9]. Studies have found that endoscopic surgery for LDH significantly reduces the reoperation rate, with recurrence rates ranging from 2.4 to 8.5% [10]. However, PELD has several practical issues. First, it requires repeated blind puncture through the skin to reach the target which can result in iatrogenic injury, the most common of which is nerve root and dural injury [11]. Second, when the puncture is inaccurate, especially when the surgeon is inexperienced, multiple repeat punctures together with X-ray fluoroscopy are required, increasing the operation time and relevant surgery risk and exposing both medical staff and patients to X-ray radiation.

A previous study reported that the average radiation doses in PELD surgery with conventional blind puncture were: eye, 0.017 mSv; thyroid gland, 0.018 mSv; chest, 0.039 mSv [12]. Other researchers have found a strong link between radiation and cancer, with long-term low doses of radiation easily causing cancer in the thyroid, lung, and other organs [13–15]. Cancer mortality was found to increase by 0.004% for both the patient and the surgeon for each millisievert radiation dose received [16]. Researchers have confirmed the relationship between cancer and the radiation dose received by bone surgeons and related physicians, and intraoperative X-ray radiation is unavoidable during PELD surgery [17]. As a result, reducing the frequency of X-ray fluoroscopy is critical to reducing the amount of radiation exposure to sensitive body parts. To reduce the frequency of X-ray fluoroscopy and radiation exposure during PELD surgery, precise positioning and puncture techniques are critical. Thus, exploring new puncture methods to reduce the number of X-ray fluoroscopy and puncture and improve precise positioning has always been a major concern for minimally invasive spinal surgeons, especially the junior surgeons.

Using the geometry principle of the symmetry of isosceles triangles (Fig. 2), the body surface-assisting puncture device clarifies the puncture route, thus reducing the difficulty of puncture and improving the puncture accuracy, as well as reducing the radiation dose. It has a simple structure, is inexpensive, easy to use, and can be used repeatedly, making it suitable for PELD surgery. The use of the body surface-assisting puncture device in PELD surgery significantly reduces the radiation dose during X-ray fluoroscopy, puncture times, and operation time; moreover, it does not increase the surgical complications and treatment cost, is suitable for all hospitals and spine surgeons, especially in small hospitals, thus demonstrating significant benefits.

The present results showed that the use of the body surface-assisting puncture device in patients in the locator group allowed the precise targeting of the preoperatively determined puncture path to the puncture point, thus reducing the radiation dose during X-ray fluoroscopy compared with the control group. The puncture success rate was 100% with no increase in surgical complications. Preoperative puncture path planning is critical to improving the puncture success rate. The distance between the skin on the median spinous process line and the anterior wall of the spinal canal of the target vertebral body was determined using an accurate calculation using imaging data before surgery. The depth of the needle puncture was calculated using a trigonometric function, which effectively avoided the disadvantages of a too-deep puncture into the abdominal cavity and insufficient puncture for multiple perspective adjustments. The preoperative quantification of the puncture angle and puncture depth is beneficial to improve the surgeon’s safety and confidence.

The accuracy of preoperative positioning is influenced by a variety of factors, including the performance of fluoroscopic equipment, the resolution of fluoroscopic images, and the experience of physicians [18]. The latter is particularly important for precise positioning and puncture but presents difficulties for junior surgeons who require practice, which can lead to numerous complications. The clinical application of the body surface-assisting puncture device in preoperative puncture-path planning in our hospital demonstrated that this device significantly reduced radiation dose during X-ray fluoroscopy, as well as the number of punctures and operation times without increasing surgical complications.

PELD for junior surgeon requires a learning curve of about 60–70 patients, whereas it has a notoriously steep learning curve, the precision positioning and puncture are significant challenges in the early stages of learning [19, 20]. Wang et al. [7] conducted a comparative analysis of the learning curve of PELD for junior surgeons and found that the technology requires significant time to learn with initial slow progress. The precise puncture positioning plan of this device is helpful to reduce the difficulty for young doctors to learn PELD and build up their confidence.

However, this new body surface-assisting puncture device has some limitations. While the ideal condition with the body surface-assisting puncture device is a single successful puncture, some cases require at least two to three punctures. Due to technical reasons and flaws in the body surface-assisting puncture device, such as the thickness of the base itself, the radian of the human waist, measurement error, and accuracy of height adjustment of the body surface-assisting puncture device, specific errors in the puncture path planning can occur. These errors are bound to impact the puncture and operation; thus, improvements and upgrades are still required. Despite these limitations, a retrospective clinical study found that this body surface-assisting puncture device reduced the radiation dose during intraoperative X-ray fluoroscopy, puncture times, and operation time, indicating that it could be useful in PELD surgery.

## Conclusion

The new body surface-assisting puncture device performed well in PELD surgery, significantly reducing the number of punctures and X-ray fluoroscopy radiation dose as well as shortening the operation time without increasing surgical complications. Moreover, it is cheap, easy to operate, suitable for all hospitals and spine surgeons, especially in small hospitals, and does not increase the treatment cost for patients. It is a new and useful minimally invasive spinal surgical puncture instrument that deserves to be improved and promoted, and has demonstrated practical utility.

## Data Availability

The datasets used and/or analysed during the current study are available from the corresponding author on reasonable request.

## Abbreviations

PELD: percutaneous endoscopic lumbar discectomy
LDH: lumbar disc herniation
